# Urban-rural residence location and cancer-specific mortality among colorectal, lung and ovarian cancer patients: a nationwide retrospective cohort study from Lithuania

**DOI:** 10.2340/1651-226X.2025.44346

**Published:** 2025-11-18

**Authors:** Rūta Everatt, Birutė Brasiūnienė, Ieva Vincerževskienė, Birutė Intaitė, Saulius Cicėnas, Ingrida Lisauskienė

**Affiliations:** a Laboratory of Cancer Epidemiology, National Cancer Institute, Vilnius, Lithuania; b Department of Biobank, National Cancer Institute, Vilnius, Lithuania; c Faculty of Medicine, Vilnius University, Vilnius, Lithuania; d Laboratory of Clinical Oncology, National Cancer Institute, Vilnius, Lithuania; e Department of Gynaecologic Oncology, Center of Surgical Oncology, National Cancer Center of Vilnius University Hospital Santaros Clinics, Vilnius, Lithuania; f Department of Thoracic Surgery and Oncology, Center of Surgical Oncology, National Cancer Center of Vilnius University Hospital Santaros Clinics, Vilnius, Lithuania

**Keywords:** Cancer mortality, urban-rural differences, colorectal cancer, ovarian cancer, lung cancer

## Introduction

Survival from cancer has improved considerably over the last decades in most countries of Europe, including Lithuania [1, 2]. However, cancer remains the second leading cause of death in Europe (22%) and Lithuania (18%) after circulatory diseases (32% and 53%, respectively) [3]. Together with Bulgaria, the Slovak Republic, Czechia, Croatia, Poland and Romania, Lithuania has some of the lowest estimated 5-year survival rates across the 11 cancer sites, suggesting there is room for improvement [4]. Age-standardised 5-year survival was respectively 57%, 10%, and 35% for colon, lung, and ovarian cancer in Lithuanian patients, compared to 67%, 19%, and 46% in Norway.

Although citizens in countries with a universal healthcare system should have equal access to healthcare, previous studies suggest that not all patients benefit equally from the improvements in diagnostics and treatment of cancer, and disparities in cancer survival have been observed in many countries [5–9]. Lower survival has been detected in cancer patients who live in rural settings, suffer material or social deprivation, or have a low income or education [4, 9–11]. The magnitude of inequalities varies by country and over time: they are generally greater in Baltic/Central/East Europe and smaller in southern Europe [12]. Living in rural areas has been associated with less likelihood to be referred and have surgery, less timely initiation of treatment, poorer-quality care, challenges with transport, lower health literacy, and higher prevalence of risky behaviours that influence cancer risk, treatment effectiveness, and survival [13]. As diagnostics, treatment, cancer care options, and survival are constantly improving, it is important that changes do not have negative effect on patients from more deprived rural areas. In order to characterise patient groups that are potentially disadvantaged, and inform health policy administrators on quality improvements, knowledge on urban-rural disparities in cancer survival is required.

The aim of this study was to examine the effect of rural location of residence on cancer-specific mortality rates in patients with colorectal, lung, and ovarian cancer in Lithuania. We also sought to identify factors that may explain observed urban-rural disparities.

## Patients/material and methods

This retrospective cohort study was performed using data from patients with cancer diagnosed between 2013 and 2015, and identified from the Lithuanian Cancer Registry (LCR). The LCR covers the entire population of the Republic of Lithuania and contains information on the date and methods of diagnosis, cancer characteristics (tumour type, histology, stage at diagnosis, prior cancers), and date and cause of death, as well as demographic data (age at the time of diagnosis, sex, location of residence). For the present study, cancer codes C18-C21, C34, and C56 of the ICD-10 (International Statistical Classification of Diseases, 10th Revision) were used for colorectal, lung, and ovarian cancer, respectively. We excluded individuals with prior cancer diagnosis (except for non-melanoma skin cancer), with a diagnosis based on death certificate, age < 25 years and > 80 years, no histological confirmation, and Stage IV or unknown (Supplementary Figure 1). The final number of participants included in the current analysis was 3,478.

Cancer specific deaths were defined as those with an underlying cause of colorectal, lung, or ovarian cancer (ICD-10 codes C18-21, C34 or C56). Information on receipt of cancer treatment (surgery, systemic cancer therapy, and radiotherapy), as well as on other health-related factors, was collected from the National Health Insurance Fund (NHIF) database. Comorbidity was calculated as the Charlson Comorbidity Index (CCI), taking into account comorbidities during the 1-year period prior to diagnosis [14].

The primary exposure of interest was the location of residence: urban (≥3,000 population), rural (<3,000 population). We performed crude and adjusted Cox proportional hazards models to estimate associations of cancer-specific mortality rates with living in rural versus urban areas. Results were expressed as hazard ratios (HRs) and 95% confidence intervals (CIs). The time scale was the time since diagnosis, with follow-up starting at the date of diagnosis and ending at the date of death or December 31, 2020.

The adjusted Model 1 included age at diagnosis (25–50, 51–65, 66–80 years), sex and comorbidity (CCI: 0, 1, 2, 3+). We added as covariate stage at diagnosis (Model 2) and then surgery, systemic therapy, and radiotherapy treatment (Model 3). Covariates were included based on previous evidence for their potential association with the exposure and/or the outcome (Supplementary Figure 2). We tested the proportional hazards assumption for individual covariates and globally using statistical assessment of Schoenfeld residuals. There was no evidence that the proportional hazards assumption was violated for the residence location variable for any type of cancer. There was an indication of violation of proportionality for some of covariates. These variables were included as strata in the models.

All analyses were performed using STATA/IC, 11.0 by STATA software (Stata Corporation, College Station, Texas, USA). All statistical tests were based on two-sided probability, and, if less than 0.05, they were considered statistically significant.

## Results

Table 1 shows the distribution of 1,961 colorectal, 974 lung, and 543 ovarian cancer patients by urban and rural residence location. There were no differences in age or comorbidity distribution among urban and rural individuals. Among rural patients more cancers were Stage III at diagnosis compared to urban cancer patients. Furthermore, a lower percentage of rural lung cancer patients compared to urban patients received surgery or chemotherapy treatment. In total, 1,848 subjects died during the follow-up, including 1,547 cancer-specific deaths. The mean follow-up time after diagnosis was 4.1 years, the maximum was 8.0 years.

**Table 1:** Patients’ characteristics by their residence location.

	Colorectal cancer			Lung cancer			Ovarian cancer		
	Total	Urban	Rural	Total	Urban	Rural	Total	Urban	Rural
Total, *N*	1961	933	533	974	354	380	543	393	150
**Age at diagnosis (years)**									
25–50	166 (8.5)	125 (8.8)	41 (7.7)	66 (6.8)	44 (7.4)	22 (5.8)	149 (27.4)	112 (28.5)	37 (24.7)
51–65	751 (38.3)	563 (39.4)	188 (35.3)	466 (47.8)	265 (44.6)	201 (52.9)	233 (42.9)	170 (43.3)	63 (42.0)
66–80	1044 (53.2)	740 (51.8)	304 (57.0)	442 (45.4)	285 (48.0)	157 (41.3)	161 (29.6)	111 (28.2)	50 (33.3)
**Sex**									
Men	950 (48.4)	686 (48.0)	264 (49.5)	820 (84.2)	478 (80.5)	342 (90.0)	-	-	-
Women	1011 (51.6)	742 (52.0)	269 (50.5)	154 (15.8)	116 (19.5)	38 (10.0)	543	393	150
**Stage**									
I	436 (22.2)	337 (23.6)	99 (18.6)	147 (15.1)	105 (17.7)	42 (11.0)	152 (28.0)	115 (29.3)	37 (24.7)
II	729 (37.2)	532 (37.2)	197 (37.0)	255 (26.2)	154 (25.9)	101 (26.6)	52 (9.6)	37 (9.4)	15 (10.0)
III	796 (40.6)	559 (39.1)	237 (44.5)	576 (58.7)	335 (56.4)	240 (62.4)	339 (46.6)	241 (61.3)	98 (65.3)
**Surgery**	1786 (91.1)	1296 (90.8)	490 (91.9)	507 (52.0)	335 (56.4)	172 (45.3)	452 (83.2)	330 (84.0)	122 (81.3)
**Chemotherapy**	832 (42.4)	598 (41.9)	234 (43.9)	560 (57.5)	361 (60.8)	199 (52.4)	452 (83.2)	329 (83.7)	123 (82.0)
**Radiotherapy**	352 (17.9)	235 (16.5)	117 (21.9)	364 (37.4)	233 (39.2)	131 (34.5)	3 (0.6)	2 (0.8)	1 (0.7)
**CCI**									
0	1291 (65.8)	944 (66.1)	347 (65.1)	518 (53.2)	304 (51.2)	218 (56.3)	364 (67.0)	262 (66.7)	102 (68.0)
1	222 (11.3)	156 (10.9)	66 (12.4)	274 (28.1)	167 (28.1)	107 (28.2)	49 (9.0)	39 (9.9)	10 (6.7)
2	312 (15.9)	219 (15.3)	93 (17.4)	116 (11.9)	75 (12.6)	41 (10.8)	64 (11.8)	45 (11.4)	19 (12.7)
3+	136 (6.9)	109 (7.6)	27 (5.1)	66 (6.8)	48 (8.1)	18 (4.7)	66 (12.2)	47 (12.0)	19 (12.7)
**Diabetes**	260 (13.3)	191 (13.4)	69 (13.0)	52 (5.3)	42 (7.1)	10 (2.6)	42 (7.7)	37 (8.6)	8 (5.3)
**Hypertension**	1032 (52.6)	749 (52.4)	283 (53.1)	414 (42.5)	290 (48.8)	124 (32.6)	245 (45.1)	181 (46.1)	64 (42.7)
**Deaths**									
All	768 (39.2)	510 (35.7)	258 (48.4)	816 (83.8)	480 (80.8)	336 (88.4)	264 (48.6)	180 (45.8)	84 (56.0)
Cancer-specif. (% of all)	570 (74.2)	370 (72.5)	200 (77.5)	753 (92.3)	437 (91.0)	316 (94.0)	224 (84.8)	153 (85.0)	71 (84.5)

CCI: Charlson Comorbidity Index.

Data are numbers (%), unless stated otherwise.

Table 2 shows the urban-rural differences in mortality rates before and after adjustment for covariates. Univariate analysis revealed significantly higher cancer-specific mortality rates for those living in rural areas compared with urban areas for all three cancer types. For colorectal cancer, when adjusted for age, sex and comorbidity, the result remained almost unchanged, whereas after further adjustment for stage the estimate was reduced. Accounting for treatment had hardly any effect on mortality rates in colorectal cancer patients living in rural versus urban location. Among lung cancer patients, a moderate impact after adjustment for stage was observed. After additional adjustment for cancer treatment, the mortality difference was substantially reduced and became insignificant. In ovarian cancer patients, the rural location was associated with higher mortality rates, and these results were robust to adjustment for age, comorbidity, stage, and cancer treatment.

**Table 2:** Cox regression analyses of cancer-specific mortality in relation to rural versus urban residence location among colorectal, lung and ovarian cancer patients in Lithuania. Sensitivity analyses for available covariates.

Cancer type	Unadjusted HR (95% CI)	Model 1^a^ HR (95% CI)	Model 2^b^ HR (95% CI)	Model 3^c^ HR (95% CI)
**Colorectal**				
Urban	1	1	1	1
Rural	1.61 (1.35; 1.91)	1.58 (1.33; 1.88)	1.47 (1.24; 1.75)	1.48 (1.24; 1.76)
*p*-value^d^	< 0.0001	< 0.0001	< 0.0001	< 0.0001
**Lung**				
Urban	1	1	1	1
Rural	1.37 (1.18; 1.58)	1.33 (1.15; 1.54)	1.27 (1.10; 1.47)	1.13 (0.97; 1.31)
*p*-value^d^	< 0.0001	< 0.0001	0.001	0.12
**Ovarian**				
Urban	1	1	1	1
Rural	1.38 (1.04; 1.82)	1.45 (1.09; 1.92)	1.43 (1.08; 1.91)	1.42 (1.07; 1.89)
*p*-value^d^	0.03	0.01	0.01	0.02

HR: hazard ratio; CI: confidence interval.

a Model 1 includes age group, sex and Charlson Comorbidity Index (CCI).

b Model 2 includes age group, sex, CCI and stage.

c Model 3 includes age group, sex, CCI, stage, surgery, chemotherapy and radiotherapy treatment (for ovarian cancer radiotherapy treatment not included due to low number of patients treated).

d For heterogeneity.

## Discussion and conclusion

We found that patients with colorectal, lung, and ovarian cancer living in rural areas had higher cancer-specific mortality rates compared to those living in urban areas. Our results show urban-rural inequality regarding receiving surgery or chemotherapy treatment among lung cancer patients in Lithuania. Furthermore, rural lung and colorectal cancer patients were more likely to be diagnosed with later stage cancer compared to urban patients. Differential treatment and stage largely explained the urban-rural disparities in lung cancer mortality. In colorectal cancer patients, a moderate mediating effect of stage was observed. Results for ovarian cancer held true after controlling for factors included in the analysis.

Our findings are in line with previous studies from European countries and the United States of America (US) that have reported urban-rural inequalities in cancer survival [15–18]. Disease stage, health-related lifestyle behaviours, co-morbidities, and treatment have been reported as key factors contributing to the differences in cancer mortality rates by residence location, although the mediating effect of these factors varied across cancer sites and studies [16].

We found, that tumour stage at diagnosis contributed to urban-rural disparities in colorectal cancer mortality. Likewise, Lejeune et al. found tumour stage at diagnosis to be the primary reason for disparities in colorectal cancer survival by socio-economic position, possibly due to higher rates of screening participation and better access to diagnostic services among advantaged people [17]. However, in our study adjusting for stage only partly explained the mortality gradient by urban-rural status, thus, determinants other than those captured may play a role. Our results among lung cancer patients are in agreement with previous studies where rural residence was associated with worse survival outcomes [18]. We found that adjustment for cancer treatment and stage substantially reduced the differences and HR became insignificant. Thus, the urban-rural differences in lung cancer mortality could probably be explained by lack of receipt of cancer treatment and inequality in stage. We also found that women with ovarian cancer who live in rural settings had increased HRs compared to those living in urban areas, similar to previous studies [15]. It has been demonstrated that ovarian cancer patients from more deprived areas or rural areas are less likely to receive surgery or systemic cancer therapy, experience long secondary care delays, and wait longer to undergo treatment [19, 20]. However, in the present study neither receipt of cancer treatment or other factors (stage, age, and comorbidity) could explain the observed urban-rural differences in ovarian cancer mortality rates. This suggests that factors not estimated in this study such as health seeking behaviour, health related life style and access to health care (distance to care, shortage of specialists in rural settings, etc.) cannot be ruled out.

A major strength of this study is the use of a population-based cancer registry linked to the NHIF database. This linkage provided detailed and free of recall bias information on cancer diagnoses and clinical factors such as cancer stage, cancer therapies, and comorbidities. The main limitation of the study is the relatively low number of cancer deaths in this cohort of cancer patients and therefore limited statistical power for subgroup analyses. We had no information on lifestyle and socioeconomic factors such as smoking, body mass index, diet, physical activity, income, education, cohabitation status, frailty, or the severity of comorbidities, and their role in certain cancer progression has been shown [21–23]. Thus, residual confounding by these and other unmeasured variables (depicted in Supplementary Figure 2), may be a possible explanation for the observed urban-rural differences.

In conclusion, residence in rural areas was related to increased cancer-specific mortality rates in colorectal, lung, and ovarian cancer patients. Among lung cancer patients, stage and receipt of cancer treatment largely contributed to observed differences in mortality rates. In colorectal cancer patients, disparities are partly explained by differences in stage at diagnosis. This emphasises the importance of improvements in early detection and also optimal treatment among rural cancer patients. However, there are still determinants of the urban-rural disparities in cancer patients that were unexplained. Differences may be attributable to unmeasured factors that need to be further investigated.

## Supplementary Material



## Data Availability

The data that support the findings of this study are not publicly available due to privacy or ethical restrictions but are available from the corresponding author on reasonable request and with permission of Vilnius Regional Biomedical Research Ethics Committee (VRBREC).
