# Supplementary material for: Urban-rural residence location and cancer-specific mortality among colorectal, lung and ovarian cancer patients: a nationwide retrospective cohort study from Lithuania
Source: Acta Oncol. 2025 Nov 18;64:44346. doi: 10.2340/1651-226X.2025.44346 (PMC12640107; doi:10.2340/1651-226X.2025.44346)
Supplement: Supplementary file 1 [file AO-64-44346-s1.pdf]

Supplementary material has been published as submitted. It has not been copyedited, or typeset by Acta Oncologica

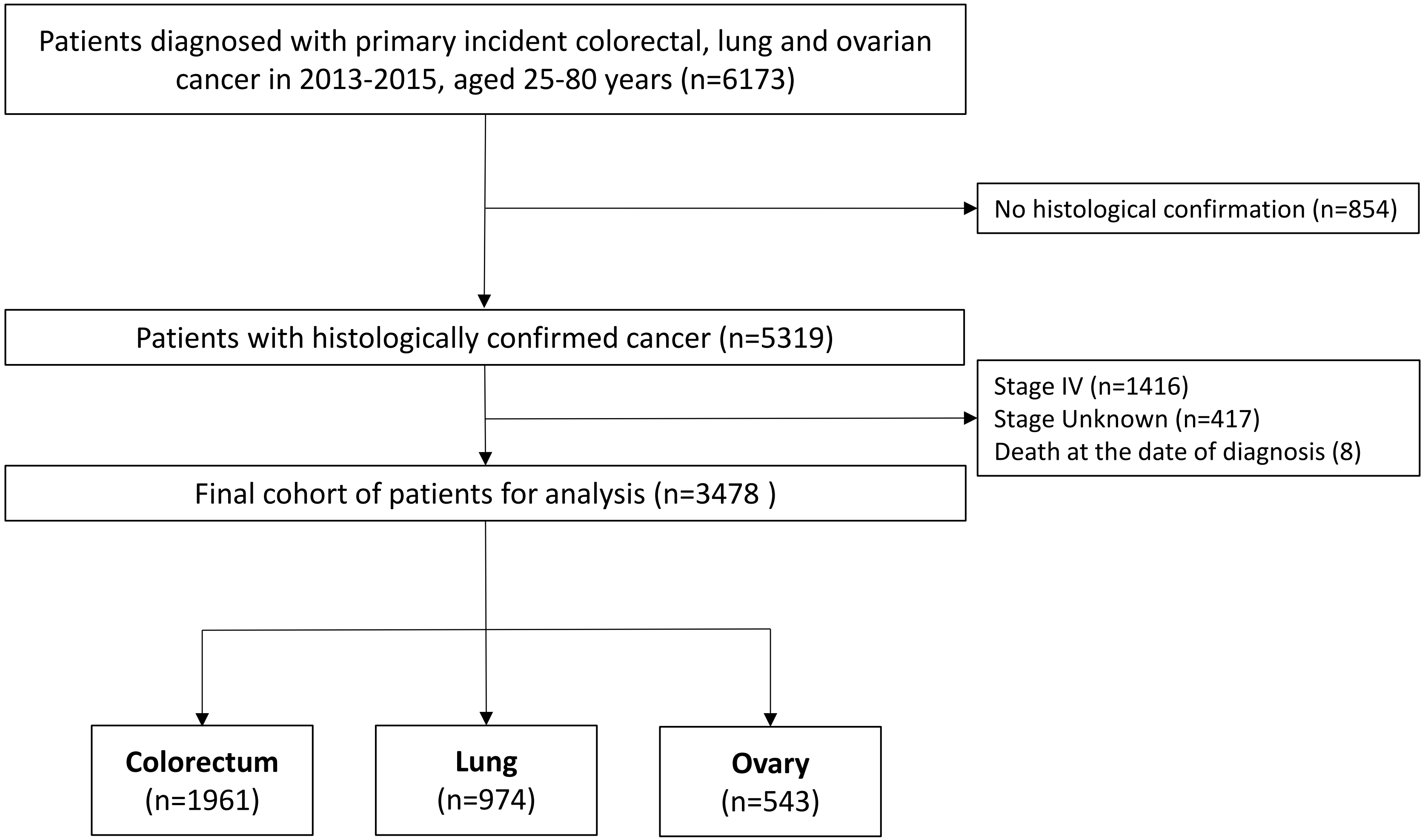

Supplementary Figure 1. Flow diagram of data management and selection of subjects for the study.

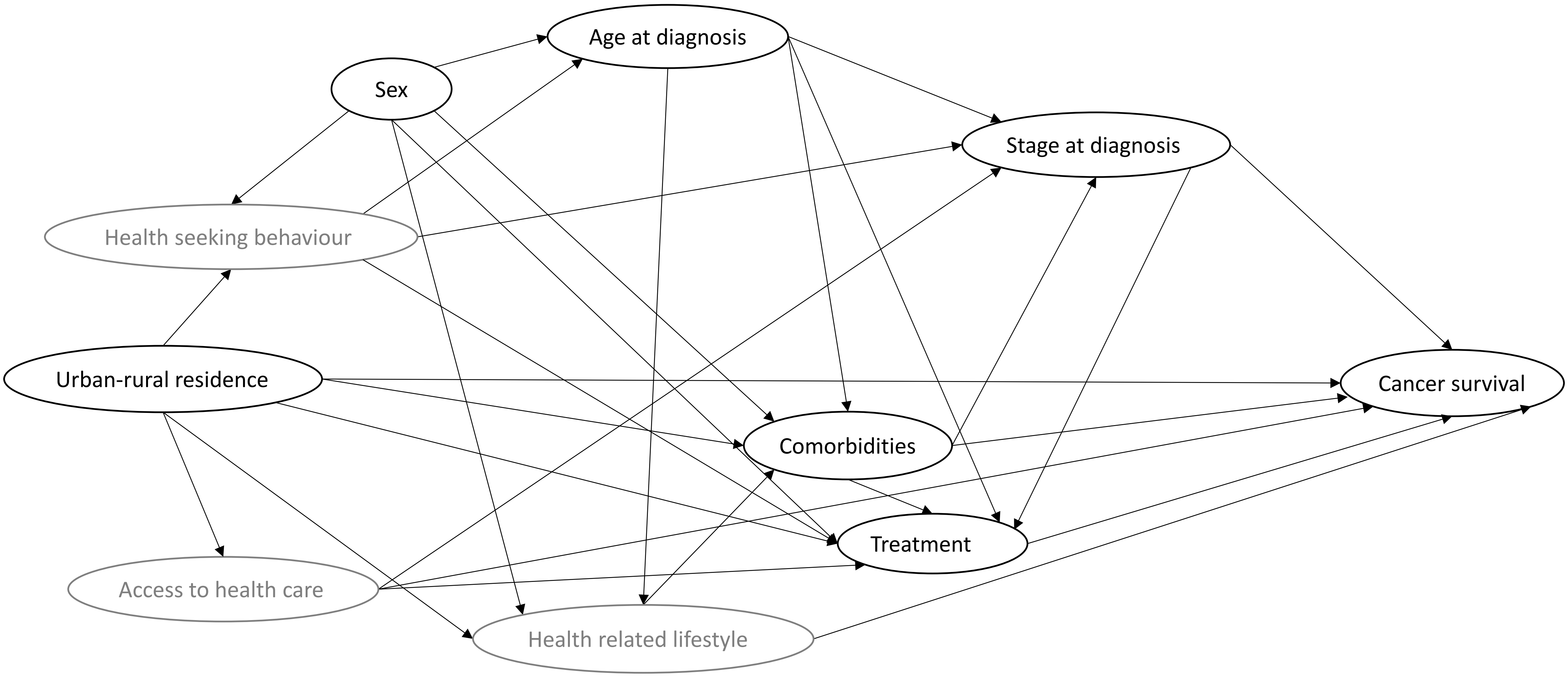

Supplementary Figure 2. Direct acyclic diagram (DAG) depicting assumed causal relationships between residence location and survival status in cancer patients. (Unmeasured variables shown in grey).
